# Prevalence and burden of self-reported blindness and low vision for individuals living in institutions: a nationwide survey

**DOI:** 10.1186/1477-7525-3-27

**Published:** 2005-04-25

**Authors:** Antoine Pierre Brézin, Antoine Lafuma, Francis Fagnani, Mounir Mesbah, Gilles Berdeaux

**Affiliations:** 1 Hôpital Cochin – Service d'ophtalmologie – Université Paris 5 – 27 rue du Faubourg Saint-Jacques F-75679 Paris cedex 14, France; 2 Cemka, 43, Boulevard du Maréchal Joffre, F-92340 Bourg-la-Reine, France; 3 Université Pierre et marie Curie, 175, rue du Chevaleret, F-75013 Paris France; 4 Gilles Berdeaux, Alcon France, 4 Rue Henri Sainte Claire Deville, F-92563 Rueil-Malmaison Cedex, France

**Keywords:** Blindness, Low vision, Handicap, Incapacity, Dependency

## Abstract

**Background:**

The prevalence of self-reported low vision (LV) and blindness, and their associated disabilities, handicaps and socio-economic consequences for individuals living in institutions are poorly documented.

**Methods:**

2,075 institutions were selected at random and eight individuals were picked at random from the list of residents. Three groups of individuals were defined: blind, LV, and a control group (CG). These were compared after adjustment for age and co-morbidities. Of the 15,403 individuals, 14,603 interviews (94.9%) were completed.

**Results:**

The prevalence of blindness was 1.6% and the LV 13.4%. Blind individuals needed assistance more often (OR: 2.65 to 11.35) than CG members while the assistance required by LV individuals was similar to that for the CG. Blind individuals required institution adaptation (building and furniture changes) more often than the CG. Blind (57.9%) and LV individuals (35.4%) were more often registered for social allowances. Monthly social allowances were EUR 86 higher for blind than LV individuals. Monthly family incomes were found to be similar between the three groups (from EUR 782 to 797). Social and demographic data, institution description, income, handicaps, disabilities, social allowances and details of daily activities were collected interviews

**Conclusion:**

The results demonstrate the impact of self-reported blindness and LV on daily life for patients living in institutions.

## Background

Aging creates policy challenges for most developed countries that increase pressure on social and care systems [1,2]. An institution is often the final care facility when older people have too many disabilities. In developed countries, it was estimated that 6.5% of individuals older than 65 years lived in institutions in 1994 [3] with an annual growth rate of 0.8%. The number of individuals in French institutions will show an increase of 56.3% by 2020 mainly due to the aging of the population. In 2000, institutionalization represented 0.62% to 2.71% of the GDP (Gross Domestic Product) in OECD countries. Increases of up to 69.5% in the number of institutionalized individuals are expected by 2020 [4].

In 2001, according to the World Health Organization (WHO), visual impairment was responsible for 2,286,000 DALY (disability adjusted life years) in the high income countries. [5]. The prevalence of blindness and low vision (LV) for people living in institutions has never been estimated at a national level as far as we are aware. The use of registers to estimate the prevalence of blindness is controversial since a high proportion of blind individuals are not registered [6-9]. Studies were conducted in institutions [10-12], but never from a representative nationwide sample. None of them documented the level of disability linked to visual impairment. Visual impairment prevalence rates varied from 7.4% to 23.0%, this range being mainly explained by different definitions of visual impairment. Since blindness and/or LV could be one of the impairments leading to institutionalization in elderly individuals, the comparison of prevalence in the community and in institutions is a key issue.

The social and economic consequences of blindness (disability, dependency and need for assistance) have never been evaluated, in France, at a national level with a representative sample of individuals living in institutions. This information is important for several reasons: (1) institutions have to provide the right level of assistance for each different type of impairment, blindness being one of them; (2) a handicap needs to be indemnified (directly or indirectly) and therefore its economic burden have to be estimated to determine the level of social allowances; (3) macro-economic consequences of blindness need to be estimated to determine how much should be budgeted and invested to care for this handicap at a national level in the long term.

The present survey had three aims: (1) to estimate the prevalence of self-reported blindness and LV in French individuals living in institutions, on a national basis; (2) to study the consequences of self-reported blindness and LV, focusing specifically on disabilities (restricted ability, or inability to perform the activities of daily living) and handicaps (restricted ability, or inability to fulfill desired social roles); (3) to collect information on the economic consequences of income level and social allowances.

## Methods

This survey was conducted at the request of the French State which also provided the finance. The data were gathered by INSEE (Institut National de la Statistique et des Etudes Economiques) which is the State agency responsible for executing regular national census surveys. This survey complied with all existing national regulations, including personal data privacy and access. The database was subsequently made available to researchers for secondary analyses.

The methodology of this survey has already been described elsewhere[13]. Cluster sampling was used.

2,075 institutions were randomly selected from French Health Ministry files (day care centers were excluded). French institutions are classified into four types according to the nature of the residents: children with handicaps, adults with handicaps, the elderly and psychiatric patients. . The sample was stratified by 4 main categories of institutions (Table 1). The probability of an institution being selected was inversely proportional to the number of institutions in the category, and was proportional to the number of beds. Fifty-seven had to be replaced: 37 had no residents, 18 no longer existed, and the survey was not technically possible in seven centers. One hundred and fifty-five (7.5%) of the institutions refused to participate. Refusal rates varied by type of institution: 6.5% in centers for handicapped children, 4.5% in centers for handicapped adults, 4.5% in elderly care homes and 17.0% in psychiatric centers. The most frequent reasons for refusal to participate were lack of time (22.7%), the non-compulsory (this survey was disconnected from the national census) nature of this INSEE survey (10.7%), lack of staff to help the interviewer (7.3%), disturbance of the residents (5.3%), institution being restructured (3.3%), violation of privacy (2.7%), too many surveys (2.7%), and beliefs that the questionnaire was not adapted to the residents (2.7%).

**Table 1:** Experimental design. Sampling plan. Category description

	**Institution (Sampling frame)**		**Individuals (Sampling elements)**	
	
	**Population**	**Sampling rate**	**Population**	**Sampling rate**
Children institution	1,206	34.2%	48,398	6.8%
Adult institution	2,405	18.7%	82,852	4.3%
Psychiatric institution	394	79.2%	70,932	3.5%
Elderly care home	7,414	12.0%	490,963	1.4%

Children institution included intellectual, motor and sensorial handicap. Adult institution included individuals able to work outside, inside the institution, need for medical assistance and other type of institution. Psychiatric institution included mental disease specialized, psychiatric, and other type of hospital. Elderly institution included various types of institution, private, public, charity, short and long term, with no, mild and high medical activities.

Of these institutions, 15,403 individuals were taken at random by the interviewers from the resident lists (eight per institution) and 14,611 interviews (94.9%) were performed. The analysis was performed on the 14,603 patients with documentation of handicap (eight interviews were stopped before the handicap was documented).

The questionnaire is available from the INSEE upon request by researchers.

No exclusion criteria were specified (e.g. age, cognitive functioning, etc ..) and proxy responded when required by the health status of the interviewed. The presence of handicap was identified by the following initial "yes/no" question: "In everyday life, are you faced with either physical, sensorial, intellectual or mental difficulties (resulting from an accident, a chronic disease, a problem at birth, a disability, aging ...) ?" Independent of the answer, the following question was asked to list handicaps: "What kind of difficulties, disabilities, or other health problems do you suffer from?" All the answers were written down without any alteration. If, during the rest of the questionnaire, the interviewers came across diseases or other health problems not mentioned earlier, they were also reported. The declared morbidity was then medically coded by experts in this field, using ICD classification. More details on coding, quality control, training are available from the INSEE upon request.

For the purpose of this study, blindness and LV were defined based on individual declaration, independent of any medical information. At the beginning of the interview, individuals were asked whether they experienced physical, sensorial, intellectual or mental difficulties in their daily life, and the nature of the difficulties. During questioning on incapacity, the incapacity had to be related to a handicap; this provided a second chance to capture data on handicaps. Three closed questions dedicated to vision were asked during the interview: (1) Do you have trouble reading newspapers, books, etc ... with your glasses, if you wear any? (2) Do you have trouble recognizing the features of someone standing four meters from you (with eyeglasses or contact lenses, if you usually wear any)? (3) Would you say you are completely blind (light perception at best), partially blind (still have form perception) or visually impaired? Data were collected as free text and experts in medical coding made post-hoc classification of declared disease. Question 1 (near vision) and question 2 (distant vision) were used to identify individuals with vision troubles. Only the latter had to answer to question 3. According to the previous answers, individuals were classified into one of the following groups: (1) blind; (2) low visual acuity; (3) control (i.e. neither blind nor LV). Blind people declared only light perception. Low visual acuity people declared either severe difficulties in long or short distance vision to question 1 and 2 and form perception or visual impairment to question 3. The worst severity in terms of visual impairment (blind >LV >NVP) was always retained.

Disabilities were described according to an ordinal scale following a question started by "Can you ...?". Answers varied according to the handicap, but the general structure was as follows: "Irrelevant", "Yes, without assistance and without any trouble", "Yes without assistance but with some difficulty", "Yes, without assistance, but with much difficulty because of my physical disorders", "No, I need partial assistance", "No, I need assistance for everything", and "Will not answer or does not know". The figures presented in the tables grouped partial assistance and assistance for everything together.

Social allowances covered the following groups of items: allowance for disabled adults, compensatory allowance, income guarantee, special education allowance, housing allowance, special dependency allowance, disablement paid by the State, disablement allowance deriving from an accident at work, daily allowance paid by the French Sick Fund, allowance paid by an insurance company, military disablement allowance, and other. Details within groups were collected. Results were expressed in EUROs. Revenue was defined as the sum of incomes and social allowances.

Data were collected from October to December 1998 by 413 personal interviewers. The mean interview time was 38 minutes. A trained interviewer filled in the questionnaire at the institution using remote data entry computer software. The data included type of handicap and disabilities (if any), indexes of daily activity (Katz, Colvez and EHPA indexes [14,15]), the reasons for and duration of handicaps, social professional and family environment, individuals' social demography, institution characteristics, institution adaptations required for the handicap, mobility difficulties, household income (including social allowances), social allowances and public and private health insurance.

All analyses were conducted with SAS (SAS Institute, North Carolina) software, release 8.2. Since the three sub-groups were not comparable, adjustments were made using a weighted logistic regression for qualitative variables and a weighted analysis of variance for quantitative parameters.

Weighting factors required for national extrapolation included size of the category, the institution occupation rate (number of individuals in the institution / number of available beds) and the answer refusal rate (higher in psychiatric centers).

Populations were made comparable using the blind population as the reference. Adjustments were performed on age and number of co-morbidities since these variables were found to be linked to almost all of the parameters. Rates were adjusted by using the blind population estimates as reference: estimators of the logistic regression were applied to the co-variables estimated on the blind population.

Odds ratios (OR) were calculated by using the control population (i.e. without visual problems) as a reference. All tests were interpreted two-sided, alpha fixed at 0.05. No corrections to take into account test multiplicity were applied.

## Results

Out of 14,603 individuals interviewed (Table 2), 265 were classified as blind, 1,622 had LV and 12,716 did not claim a visual handicap. Upon extrapolation to the whole population (664,252 individuals), the corresponding prevalence figures were 10,394 blind individuals (1.56%) and 89,252 with LV (13.4%).

**Table 2:** Socio-demographic parameter and handicap description

	**Blind n = 265 (n = 10,394)**	**Low vision n = 1,622 (n = 89,252)**	**No visual problems n = 12,716 (n = 564,606)**
Age (years)	71.4	80.0	67.3
Male	30.3%	26.6%	37.5%
Entry to institution related to health	88.0%	72.8%	77.5%
Institution with medical services	44.2%	31.2%	34.5%
Time in institution (years)	9.2	5.2	5.3
Number of handicaps outside vision	1.62 (1.50)	1.99 (1.82)	1.68 (1.59)
Motor handicap	54.2% (51.0%)	73.5% (68.8%)	51.9% (57.4%)
Auditory handicap	13.9% (18.6%%)	35.0% (26.1%)	22.3% (12.4%)
Vocal handicap	6.0% (4.8%%)	5.3% (5.3%)	5.2% (5.3%)
Visceral handicap	23.4% (21.1%)	35.6% (28.8%)	23.5% (22.9%)
Cognitive handicap	42.1% (38.4%)	34.0% (36.0%)	38.5% (40.9%)
Other handicap	26.8% (18.6%)	15.7% (17.4%)	20.1% (23.7%)
Katz classification 'A'	24.4% (20.7%)	40.7% (50.6%)	47.5% (45.7%)

Katz 'A' classification: able to wash, dress, go to the restroom, go to bed and stand up, sphincter control, eat meal already prepared, alone. Number of handicaps outside vision adjusted on age (2^nd ^line, figures within brackets). Katz 'A' classification adjusted on age, and number of handicaps outside vision (3^rd ^line, figures within brackets).

On average, LV individuals were older than blind and control individuals (80.0, 71.4 and 67.3 respectively). About one-third of the individuals were male. Blind people utilized an institution with medical services more frequently than LV and control individuals. The average time spent by blind individuals is around four years longer than for LV individuals and controls.

The number of handicaps excluding vision was slightly higher in the LV group and the difference persisted after adjustment. More motor, auditory and visceral handicaps were reported by LV individuals. According to the Katz classification, blind people were able to self-wash, dress, go to the restroom as well as control elimination functions, get-up from bed, and eat without assistance less often than LV and control individuals (24.4%, 50.6% and 47.5% respectively, after adjustment for age and number of co-morbidities).

Almost no difference in need for assistance was found between LV individuals and individuals who declared no problems with vision (Table 3). Blind individuals more often needed assistance to do most of their normal daily tasks (Odds-ratio (OR) between 2.65 and 11.35). This mainly concerned the following activities: shopping (OR: 11.35), getting a drink (10.68), using a lift (7.63), climbing steps (7.19), mobility on one level (7.09) and walking outside (7.02).

**Table 3:** Assistance and burden for the care giver according to level of vision impairment

**Need assistance**	**Blind**	**Low vision**	**No visual problems**	**P-Value**
Self-washing	66.2% (2.65)	36.8% (0.79)	42.5% (Ref)	<0.0001
Dressing	71.1% (3.39)	36.3% (0.79)	42.0% (Ref)	<0.0001
Cutting food	52.0% (4.43)	18.8% (0.95)	19.7% (Ref)	<0.0001
Help yourself to a drink	74.9% (10.68)	22.7% (0.99)	22.9% (Ref)	<0.0001
Drinking and eating once food is ready	32.0% (3.92)	7.5% (0.67)	10.7% (Ref)	<0.0001
Going alone to the restroom	53.5% (3.91)	17.6% (0.72)	22.8% (Ref)	<0.0001
Getup from a bed	52.2% (2.87)	23.3% (0.80)	27.5% (Ref)	<0.0001
Getup from a seat	41.8% (2.70)	16.4% (0.74)	21.0% (Ref)	<0.0001
Mobility on one level	61.6% (7.09)	16.9% (0.90)	18.5% (Ref)	<0.0001
Climbing steps	77.4% (7.19)	34.6% (1.11)	32.3% (Ref)	<0.0001
Using a lift	81.8% (7.63)	35.1% (0.92)	37.1% (Ref)	<0.0001
Walking outside	87.3% (7.02)	53.4% (1.17)	49.5% (Ref)	<0.0001
Shopping	97.0% (11.35)	76.9% (1.16)	74.1% (Ref)	<0.0001

Adjusted according to the blind French population on age, number of handicaps and size. Odds-ratio within brackets adjusted on age and number of handicaps outside vision.

Institution adaptations (building and furniture changes) were more often required for blind people than LV individuals and controls (54.5%, 39.4%, 38.0%, respectively) (Table 4). Globally the level of unmet needs concerning institution adaptation was rather low (around 1%), although slightly higher for blind individuals (5.8%). Four items were mainly concerned: bed, bathroom, restrooms and ramps.

**Table 4:** Institution adaptation, equipment according to the vision status

	**Blind**	**Low vision**	**No visual problems**	**P-Value on needs**
Institution adaptation (Need – Unmet need)	54.5% – 5.8%	39.4% – 1.3%	38.0% – 1.1%	<0.0001
Restrooms	29.9%	22.1%	20.7%	<0.0001
Bathroom	31.3%	19.2%	20.3%	<0.0001
Tables	23.6%	11.2%	14.3%	<0.0001
Seats	24.2%	11.6%	12.8%	<0.0001
Bed	46.5%	25.2%	28.3%	<0.0001
Ramps, etc.	25.0%	17.5%	18.6%	<0.0001
Devices to open doors, etc.	1.8%	2.1%	1.6%	<0.0001
Other pieces of furniture	5.1%	2.5%	1.8%	<0.0001

Adjusted according to the blind French population on age, number of handicaps. First figure : Needed institution adaptation; Second figure: unmet need

The need for medical devices increased from individuals who declared no problems with vision to people who declared themselves to be blind (38.9% – no visual trouble -, 48.5%- LV – and 64.8% – blind -) (Table 5). The level of unmet needs was very low (less than 2%). Wheel chairs were by far the most frequently required medical devices.

**Table 5:** Devices dedicated to blindness

	**Blind**	**Low vision**	**No visual problems**	**P-Value**
Devices (purchased- Unmet Need)	64.8% – 1.5%	48.5% – 1.0%	38.9% – 0.6%	<0.0001
Stick	5.6%	18.0%	12.7%	<0.0001
White stick	11.5%	2.0%	0.0%	<0.0001
Walking aids	1.2%	7.0%	6.2%	<0.0001
Wheel chair	36.5%	19.7%	20.3%	<0.0001
Dog	0.2%	0.2%	0%	0.98
Optical assistance	0.2% – 3.1%	8.6% – 8.5%	0.5% – 0.8%	<0.0001
Computer interface	0.3% – 3.4%	0.1% – 0.4%	0% – 0%	<0.0001
Software adapted for blindness	1.0% – 4.5%	0.1% – 1.0%	0% – 0%	<0.0001
Tape recorder	2.0% – 1.6%	0.3% – 1.1%	0% – 0%	<0.0001

Adjusted according to the blind French population on age, number of handicaps. First figure : purchased devices; Second figure : unmet needs.

Only one-third of the LV group and less than 60% of blind individuals received social allowances (Table 6). The rate of private insurance was slightly higher in LV individuals. Although statistically significant, the difference of monthly average allowances between blind and control individuals was quantitatively small (EUR 80). Finally, no differences were found in monthly average income between the three groups.

**Table 6:** Working activities, social allowances, copayment and revenue according to the vision status

	**Blind**	**Low vision**	**No visual problems**	**P-Value**
Social allowance	57.9%	35.4%	37.0%	<0.0001
Private insurance	54.9%	61.3%	58.9%	<0.0001
Monthly average allowances (EUR)	254	168	174	<0.0001
Total monthly average income (EUR)	782	787	797	<0.80
Total monthly average income (EUR)-Paid institution cost	116	119	121	<0.94

Adjusted according to the blind French population on age, number of handicaps and size of the household. Total monthly average income includes social allowances. EUR 1 close to US$ 1 in 2002.

## Discussion

The present analysis aimed at estimating the prevalence of self-reported blindness and LV in French individuals living in institutions and studying the disabilities related to visual impairment and its economic consequences. Since the questionnaire and the statistical techniques used in this survey was identical to the one used in a survey conducted on individuals living in the community [16], the comparison of the results between the two studies is rather straightforward.

Prevalence of self-reported blindness was 0.10% in the community and 1.56% in institutions. Respective figures were 1.94% and 13.4% for individuals with LV. The probability of being in an institution was 15.6 times higher for blind people and 6.8 times for LV individuals. These preliminary results suggest that blindness and LV might be associated with institutionalization. These relative risks need to be adjusted for the presence of other handicaps in order to confirm whether blindness and LV are independent risk factors for institutionalization.

Individuals with LV living in the community were younger (61.4 versus 80.0) than those living in institutions [16] and had less co-morbidity (1.47 versus 1.99). These differences were not found in the blind population. Mean age of blind individuals was 72.3 in the community and 71.4 in institutions. Mean number of handicaps of blind individuals was 1.58 in the community and 1.62 in institutions. This suggests that the process of institutionalization is different for blind and LV individuals. Blind people entered the institution when they were younger and they had on average a similar number of co-morbidities compared to the control group. However, the need for assistance (Katz index) is much higher for blind individuals than for controls. This suggests that blindness and its related dependency are associated with institutionalization. LV individuals entered the institution when they were older with a higher number of handicaps (after adjustment for age) than the population with no visual problems. This suggests that the association of handicaps, blindness being one of them, is linked with the institutionalization.

As expected, people living in institutions needed more assistance than those living in the community. People with no visual problems declared assistance needs that were very similar to the LV individuals. Blind individuals needed assistance more often than individuals with no visual problems (OR between 2.65 and 11.35).

Concerning institution adaptation (building and furniture changes) for the handicapped, the level of unmet needs was rather low, although one out of every 20 blind individuals had some demands that were unsatisfied. The demands of LV individuals were very similar to those of people with no visual problems. In contrast, blind people had some specific needs requiring particular institution adaptations with related costs.

57.9% of blind people living in institutions were registered for a social allowance and 35.4% of LV individuals. Concerning the monthly average allowances, the difference between LV and blind individuals was smaller for people living in institutions (EURO 86) than that calculated in the community (EURO 277). This might be explained by the fact that the financing of institutionalization includes some direct transfer from social allowances to the institution.

This study shares the same limitations as the one conducted in the community:

(1) The cross-sectional design of the survey did not permit an analysis of possible causal relationships between blindness, handicap, dependency and incapacity.

(2) The visual acuity (VA) of responders was neither measured nor controlled by an ophthalmologist and we were unable to apply the WHO classification for blindness (VA<3/60; ICD 10) [17]. The major limitation was that the primary outcome, visual impairment and blindness, was based upon interviews. There are several major problems with this. First, the validity of defining visual impairment and blindness by interview was uncertain. It was difficult to be sure that the prevalence estimates of 'visual impairment' and 'blindness' were accurate. Were they over-estimated or under-estimated? Second, there was no *post-hoc *simple medical algorithm indicating how low visual acuity and blindness were discriminated by the interview questions. Third, because both the outcome of interest (visual impairment and blindness) and risk factors (non-vision-related handicaps, need for assistance etc.) were determined by similar interview questions, the association between visual impairment and incapacity, assistance and economic consequences could be the result of reporting bias. Moreover, some patients in institutions could be severely cognitively impaired and experience significant trouble answering the questions of this survey. These problems could have generated measurement errors in both the visual impairment classification and the evaluation of disability.

The self-reported visual impairment prevalence rates we observed were very close [10-12] to those reported in other western developed countries for blind and LV individuals on subjects living in institution. At least at a national level, this gives empirical external validity to our classification of visual impairment. It is also worth commenting that we observed large differences between LV and blindness, in terms of incapacity, dependency and financial consequences. These differences persisted after adjustment on age, number of other handicaps and number of people in the household. Hence, the *post hoc *discriminant validity of blind and LV individuals seems acceptable. Therefore, because there were large differences between LV and blind individuals in terms of prevalence rates, incapacity, dependency and financial consequences, we decided to go ahead with distinguishing between the two groups. Lastly, misclassification might have occurred more frequently between LV and controls than between blind and LV individuals. The existence of a control group is important since the comparison between control and LV is a way to estimate the incapacity attributable to visual impairment, other things being equal (age, handicaps and size of household). Under the reasonable hypothesis that there is a continuum between visual impairment and severity of incapacity (as shown by this survey) misclassification should not have biased the overall burden borne by society. To summarize, the experimental design of this survey made it possible to estimate the self-declared visual impairment prevalence rates, for blind and LV persons, and their burden on society expressed as incapacity, dependency and economics."

Individuals classified as blind in this survey were those who self-declared that they did not perceive shapes. We believe that the interviewer-administered questionnaires were of sufficient quality to correctly differentiate between LV and blindness with a high probability of accuracy. Indeed in this elderly population, chronic diseases such as ARMD and glaucoma are known to account for the vast majority of ocular disorders. Lastly, access to ophthalmologists is free in France and the role of optometrists is negligible; theoretically, therefore, the conditions leading to visual impairment should have been identified medically in almost all French citizens.

## Conclusion

The quantification of disabilities associated with blindness is very important for public health decision makers and persons responsible for institutions. The later need to know the number of patients that could potentially join institutions. They also have to face tariff issues. How much social allowances do blind / LV individuals have? Are the needs (assistance, medical devices, etc ...) of blind people different from the others? Would any differences in the needs justify a dedicated tariff? From a public health point of view, aging is associated with handicaps that will increase the probability of institutionalization [3]. Most of the countries belonging to the OECD have decided to control and limit the number of people that could be institutionalized, mainly to try to control social expenses. To do so, the knowledge of risk factors (possibly including blindness and LV) of institutionalization is critical. This study provides nationwide estimates (rates and odds-ratios) of the age-adjusted disabilities associated with self-reported blindness (since blind people are older) of citizens living in institutions. Some future research should be conducted to confirm these findings, more specifically dedicated to visual impairment, including medically-validated assessment of LV and blindness.

## Authors' contributions

All authors have made substantial contributions to conception and design, acquisition of data, analysis and interpretation of data, have been involved in drafting the article or revising it and have given final approval of the version to be published.

## References

[B1] OECD (1997). Aging in OECD countries. A critical Policy Challenge. Social Policy study N° 20, Paris.

[B2] OECD (1998). Maintaining propriety in an Aging Society, Paris.

[B3] Jacobzone S, Cambois E, Robine JM (2000). Is the health of older persons in OECD countries improving fast enough to compensate for population ageing ?. OECD economic studies N° 30, OECD, Paris.

[B4] Jacobzone S (1999). Ageing and care for frail elderly persons: an overview of international perspectives. Labour market and social policy. Occasional papers N° 38 OECD, Paris.

[B5] Mathers C, Lopez A, Stein C, Ma Fat D, Rao C, Inoue M, Shibuya K, Tomijima N, Bernard C, Xu H Deaths and disease burden by cause: global burden of disease estimates for 2001 by world bank country groups. http://www.fic.nih.gov/dcpp/wps/wp18.pdf.

[B6] Robinson R, Deutsch J, Jones HS, Youngson-Reilly S, Hamlin DM, Dhurjon L, Fielder AR (1994). Unrecognised and unregistered visual impairment. Br J Ophthalmol.

[B7] Bruce IW, McKennell AC, Walker EC (1991). Blind and partially sighted adults in Britain: the RNIB survey. London: HMSO.

[B8] Walker EC, Tobin MJ, McKennell AC (1992). Blind and partially sighted children in Britain: the RNIB survey. London HMSO.

[B9] Wormald R, Evans J (1994). Registration of blind and partially sighted people. Br J Ophthalmol.

[B10] VanNewkirk MR, Weih L, McCarty CA, Stanislavsky Yuri, Keefe J, Taylor H (2000). Visual impairment and eye diseases in elderly institutionalized Australians. Ophthalmology.

[B11] Mitchell P, Hayes P, Wang JJ (1997). Visual impairment in nursing home residents: the Blue Mountains Eye Study. Med J Aust.

[B12] Tielsch JM, Javitt JC, Coleman A, Katz J, Sommer A (1995). The prevalence of blindness and visual impairment among nursing home residents in Baltimore. N Engl J Med.

[B13] Brézin A, Lafuma A, Fagnani F, Mesbah M, Berdeaux G (2004). Blindness, low vision and other handicaps as risk factors attached to institutional residence. Br J Ophthalmol.

[B14] Colvez A, Gardent H (1990). Les indicateurs d'incapacité fonctionnelle en gérontologie. Information, Validation, Utilisation. Ed CTNERHI PUF, Paris.

[B15] Katz S (1983). Assessing self-maintenance Activity of daily living. Mobility and instrumental activities of daily living. J Am Geriatr Soc.

[B16] Brézin A, Lafuma A, Fagnani F, Mesbah M, Berdeaux G Prevalence and burden of self-reported blindness, low vision and visual impairment in the community: a nation-wide survey. Arch Ophthalmol.

[B17] (1993). International statistical classification of diseases and related health problems. Tenth revision. World Health Organisation Ed World Health Organisation Geneva, Switzerland.

